# Publisher Correction: A Co_3_O_4_-CDots-C_3_N_4_ three component electrocatalyst design concept for efficient and tunable CO_2_ reduction to syngas

**DOI:** 10.1038/s41467-018-02848-2

**Published:** 2018-02-08

**Authors:** Sijie Guo, Siqi Zhao, Xiuqin Wu, Hao Li, Yunjie Zhou, Cheng Zhu, Nianjun Yang, Xin Jiang, Jin Gao, Liang Bai, Yang Liu, Yeshayahu Lifshitz, Shuit-Tong Lee, Zhenhui Kang

**Affiliations:** 1 Jiangsu Key Laboratory for Carbon-Based Functional Materials & Devices, Institute of Functional Nano & Soft Materials (FUNSOM), Soochow University, 199 Ren’ai Road, 215123 Suzhou, Jiangsu China; 2 Institute of Materials Engineering, University of Siegen, 57076 Siegen, Germany; 3 Department of Materials Science and Engineering, Technion Israel Institute of Technology, Haifa, 3200003 Israel

Correction to: *Nature Communications* 10.1038/s41467-017-01893-7; Article published online: 28 November 2017

The original HTML version of this Article omitted to list Yeshayahu Lifshitz as a corresponding author and incorrectly listed Shuit-Tong Lee as a corresponding author.

Correspondingly, the original PDF version of this Article incorrectly stated that “Correspondence and requests for materials should be addressed to X.J. (email: xin.jiang@uni-siegen.de), or to Y.L. (email: yangl@suda.edu.cn), or to S.-T.L. (email: shayli@technion.ac.il), or to Z.K. (email: zhkang@suda.edu.cn)”, instead of the correct “Correspondence and requests for materials should be addressed to X.J. (email: xin.jiang@uni-siegen.de), or to Y. Liu (email: yangl@suda.edu.cn), or to Y. Lifshitz (email: shayli@technion.ac.il), or to Z.K. (email: zhkang@suda.edu.cn)”.

This has now been corrected in the PDF and HTML versions of the Article.

