# Supplementary material for: A Co3O4-CDots-C3N4 three component electrocatalyst design concept for efficient and tunable CO2 reduction to syngas
Source: Nat Commun. 2017 Nov 28;8:1828. doi: 10.1038/s41467-017-01893-7 (PMC5705642; doi:10.1038/s41467-017-01893-7)
Supplement: Supplementary file 1 — Supplementary Information [file 41467_2017_1893_MOESM1_ESM.pdf]

Supplementary Tables

No.	2θ	FWHM	FWHM/rad	Particle size
#1	31.4	0.63°	0.0101	14.1
#2	37	0.68°	0.0119	14.3
#3	45	0.71°	0.0122	12.1
#4	59.6	0.73°	0.0127	12.4
#5	65.4	0.82°	0.0143	11.3

Supplementary Table 1 Co_3O_4 particle size derived from the XRD pattern using the Debye-Scherrer equation. Average size of Co_3O_4 NPs=12.8 nm.

Element	at%	wt%
C	39.9	33.9
N	52.5	52.
O	5.8	6.6
Co	1.8	7.5

Supplementary Table 2 Calculation of the elemental atom composition and weight composition of C, N and O in Co_3O_4-CDots-C_3N_4 from XPS data. The elemental atom composition and weight composition of C, N and O in Co_3O_4-CDots-C_3N_4 based on XPS data. Here, the content of O atom from In_2O_3 was deducted.

ECSA								
	**GCE**	**C_3N_4**	**CDots-C_3N_4**	**Co_3O_4-C_3N_4**	**Co_3O_4-CDots-C_3N_4**	**CDots**	**Co_3O_4**	**CDots-Co_3O_4**
**Full loading (the mass of each catalyst is shown in Supplementary Table 4)**								
**Cdl (mF)**	0.0072	0.040	0.043	0.042	0.045	0.0081	0.0128	0.0134
**ECSA (cm^2)**	0.18	1.82	1.95	1.91	2.05	0.37	0.32	0.34
**S_r/S_g**	2.55	25.7	27.6	27.0	29.0	5.23	4.53	4.81
**Partial loading (the mass of each catalyst is shown in Supplementary Table 4)**								
**Cdl (mF)**						0.0057	0.0066	0.0068
**ECSA (cm^2)**						0.032	0.04	0.045
**S_r/S_g**						0.45	0.57	0.64
**BET surface area**								
	**GCE**	**C_3N_4**	**CDots-C_3N_4**	**Co_3O_4-C_3N_4**	**Co_3O_4-CDots-C_3N_4**	**CDots**	**Co_3O_4**	**CDots-Co_3O_4**
**S_r (m^2/g)**		143.1	168.3	161.3	157.8	11.84	13.4	22.1
**S_r (cm^2)**		12.9	15.1	14.5	14.3	1.07	1.21	1.99
**S_r/S_g**		182	214	205	202	15.1	17.1	28.1

Supplementary Table 3 Electrochemical surface area (ECSA) and BET surface area. S_r-real surface area, S_g-geometrical surface area. S_r/S_g = roughness factor, GCE - glassy carbon electrode. The mass of catalyst used for the BET measurement is 0.1 g.

	**Full loading**							**Partial loading**		
**Catalyst / Mass**	**C_3N_4**	**CDots**	**Co_3O_4**	**CDots-C_3N_4**	**Co_3O_4-C_3N_4**	**Co_3O_4-CDots**	**Co_3O_4-CDots-C_3N_4**	**CDots**	**Co_3O_4**	**CDots-Co_3O_4**
**Total/μg**	9	9	9	9	9	9	9	0.09	0.54	0.63
**C_3N_4/μg**	9	0	0	8.91	8.46	0	8.37	0	0	0
**CDots/μg**	0	9	0	0.09	0	1.29	0.09	0.09	0	0.09
**Co_3O_4/μg**	0	0	9	0	0.54	7.71	0.54	0	0.54	0.54

Supplementary Table 4 The mass of catalyst used for the ECSA measurements. Full loading - the total mass of the catalyst combination used is 9 μg. Partial loading - loading of a single component only with the mass of this single component in the fully loaded composite combination (e.g. CDots = 1% × 9 μg, Co_3O_4 = 6% × 9 μg).

Supplementary Note 1： Calculation of Co_3O_4 nanparticles size derived from the

Debye-Scherrer equation using the XRD data. The average size of Co_3O_4 NPs derived from the XRD spectrum (**Supplementary** Fig. 2) of the Co_3O_4-CDots-C_3N_4 using the Debye-Scherrer equation

$$D = \frac{k\lambda}{B\cos\theta}$$

where, k=0.89, λ=0.154178 nm, B=FWHM rad, D=particle size

Supplementary Methods

Reaction product Analysis

The bulk electrolysis was further performed in an airtight electrochemical H-type cell with 75 mL 0.5 M $KHCO_3$ electrolyte in each chamber. For detection of gas products, hydrocarbons (CH_4, C_2H_4, and C_2H_6) were tested by a flame ionization detector (FID) with helium as the carrier gas. A thermal conductivity detector (TCD) was used to detect hydrogen, oxygen (collected from anode) and CO with nitrogen as the carrier gas. Cathodic gas sample was splitted into two aliquots for GC analysis equiped with a TCD detector. One aliquot was routed through a packed MoleSieve 5A column and another was routed through a packed HP-PLOT Q column before passing a thermal conductivity detector (TCD) for CO quantification.

The liquid products were collected from the cathode chambers after electrolysis and quantified by NMR (Bruker AVANCEAV III 400) spectroscopy, in which 0.5 mL electrolyte was mixed with 0.1 mL D_2O (deuterated water) and 0.1 μL dimethyl sulfoxide (DMSO, Sigma, 99.99%) was added as an internal standard.

N_2 absorption-desorption measurements for determination of BET surface area and pore size distribution

The BET surface area (C_3N_4, CDots, Co_3O_4, CDots-C_3N_4, Co_3O_4-C_3N_4, Co_3O_4-CDots and Co_3O_4-CDots-C_3N_4) and the pore size distribution of Co_3O_4-CDots-C_3N_4 (Fig. 1c) were determined from the adsorption isotherm of N_2 at liquid N_2 temperature (77 K) obtained using a Micromeritics ASAP 2020 instrument. Fig. 1c shows that the N_2 adsorption-desorption isotherms are Type IV isotherms with a H3-type hysteresis.[1-3] Such isotherms indicate that

initially molecules are adsorbed on the walls of the catalyst mesopores and then pore condensation follows (gas condenses to a liquid-like phase in the pore at a pressure less than the saturation pressure of the bulk liquid).[1] The Type IV isotherms have several possible hysteresis loops. The H3-type hysteresis loop is due to pore size > 4 nm[2] and also indicates the existence of plate-like particles or macro-pores[3].

The Micromeritics ASAP 2020 software implements the BJH method[4] to calculate the BET surface area and the pore size distribution from the N_2 adsorption-desorption isotherms. The surface area of Co_3O_4-CDots-C_3N_4 is 158.7 m^2 g^{-1}. The pore size is mostly in the range of 20 to 50 nm with some small pores between 2 to 4 nm (Fig. 1c inset).

H^+ Adsorption measurements

The proton (H^+) adsorption capacity of CDots, Co_3O_4, C_3N_4, CDots-C_3N_4, Co_3O_4-C_3N_4 and CDots-Co_3O_4-C_3N_4, was studied using the dialysis method applying a 5 mM HCl solution. A certain weight of catalyst (0.001 g CDots, 0.006 g Co_3O_4, 0.093 g C_3N_4, 0.5 g CDots-C_3N_4, 0.5 g Co_3O_4-C_3N_4 and 0.5 g CDots-Co_3O_4-C_3N_4,) was added to a 50 mL 5 mM HCl solution. The CDots, Co_3O_4, C_3N_4, CDots-C_3N_4, Co_3O_4-C_3N_4 or CDots-Co_3O_4-C_3N_4 solution was dialyzed using a semi-permeable membrane (MWCO 1000) in a 600 mL beaker and a 5 mM HCl (500 mL) dialysate. The adsorption of H^+ by the catalyst decreases the amount of H^+ in the catalyst compartment leading to a gradual crossing of H^+ through the semi-permeable membrane and dialyze into the electrocatalyst solution. The dialysate was stirred for predetermined time intervals and then 2 mL dialysate was taken out for analysis and the concentration of the dialysate HCl solution was determined by titrating with a 5 mM NaOH solution. The concentration of the dialysate HCl solution *vs*. time was obtained.

The single component catalysts weights were chosen to reflect the weight concentration of these catalysts in the Co_3O_4-CDots-C_3N_4 catalyst (1 wt% CDots; 6 wt% Co_3O_4; 93 wt% C_3N_4).

For single component (CDots, Co_3O_4 and C_3N_4), the amount of adsorbed H^+ ($Q_{single\text{-}component}$, mg) by the above specified weight of the single component was calculated using the Supplementary Equation 1:

$$Q_{\text{single-component}} = \frac{(C_0 - C_e)\times V}{1000/36.45} \quad (1)$$

where C_0 and C_e are the initial and temporal HCl concentrations (mg L^{-1}) respectively, V is the volume of HCl solution (500-2n mL, n is the number of temporal measurements), 36.45 is the molecular weight of HCl.

For the composites (CDots-C_3N_4, Co_3O_4-C_3N_4 and CDots-Co_3O_4-C_3N_4), the amount of adsorbed H^+ ($Q_{composte}$, mg g^{-1}) was calculated using the Supplementary Equation 2:

$$Q_{\text{composte}} = \frac{(C_0 - C_e)\times V}{1000W/36.45} \quad (2)$$

where C_0 and C_e are the initial and temporal HCl concentrations (mg L^{-1}) respectively, V is the volume of HCl solution (500-2n mL, n is the number of temporal measurements) and W is the weight (g) of composites. Note that $Q_{single\ component}$ represents the total weight of H^+ adsorbed per the fraction of single component catalyst in a Co_3O_4-CDots-C_3N_4 weighing 0.1g. The expected adsorption of a mixture of these components (Q_{SC}(CDots) + Q_{SC}(Co_3O_4) + Q_{SC}(C_3N_4) is compared to that of 0.1 g of the Co_3O_4-CDots-C_3N_4 (0.1 g $Q_{composite}$). The actual adsorption of 0.1 g composite was 1.38 mg, almost twice larger than the expected adsorption of the pure components which was 0.787 mg. This indicates that the chemical blending of the pure components to a composite catalyst has a positive synergistic effect on the H^+ adsorption.

CO_2 Adsorption measurements

CO_2 adsorption was determined by plotting the adsorption isotherm of CO_2 at 25 °C obtained using a Micromeritics ASAP 2050 instrument. A certain weight of catalyst (0.001 g CDots+0.099 g quartz wool, 0.006 g Co_3O_4+0.094 g quartz wool, 0.093 g C_3N_4, 0.1 g CDots-C_3N_4, and 0.1 g CO_3O_4-CDots-C_3N_4) was added into test chamber. The adsorption of CO_2 was performed at 25 ºC after two cycles of gas desorption. The amount of CO_2 molecules adsorbed on the catalyst increases along with the incremental pressure of CO_2, yielding the adsorption isotherm of CO_2 at 25 °C from which the adsorption capacity of the different catalysts at different pressures is determined. The weights of the single components (CDots,

Co_3O_4, C_3N_4 reflected their wt.% in the CO_3O_4-CDots-C_3N_4. The CO_2 adsorption of 0.1g CO_3O_4-CDots-C_3N_4 catalyst (0.0332 mmol) was larger by a factor of 3 than the adsorption of the individual catalyst components (0.01262 mmol) indicating a positive synergistic effect on the CO_2 adsorption.

The calculation of Faradaic efficiency

For CO,

$$\mathrm{FE} = \frac{2F \times n_{CO}}{I \times t} \times 100\% \tag{3}$$

For H_2,

$$\mathrm{FE} = \frac{2F \times n_{H_2}}{I \times t} \times 100\% \tag{4}$$

where F is the Faraday constant, n_{CO} is the moles of produced CO, and n_{H_2} is the moles of produced H_2.

Determination of the electrochemical surface area

The electrochemical surface area (ECSA) of all the electrocatalysts combinations was estimated from the electrochemical double-layer capacitance (C_{dl}). The C_{dl} was determined by measuring the cyclic voltammetry curves (CV) at different scan rates (5, 10, 25, 50, 100, 200 mV/s) under a non-Faradaic potential range. In this potential range, charge transfer electrode reactions are considered to be negligible and the current is originated solely from electrical double layer charging and discharging. The non-Faradaic potential range was identified from CV (typically a 0.1 V window centered at the open-circuit potential). All measured current in this region is assumed to be due to a double-layer charging. The charging current, i_c, is equal to the product of electrochemical double layer capacitance C_{dl}, and scan rate v, as given in the Supplementary Equation 5:[5]

$$i_c = v \times C_{dl} \tag{5}$$

A plot of i_c as a function of v yields a straight line with a slope equal to C_{dl}.

The ECSA of a catalyst sample is calculated from the double layer capacitance according

to the Supplementary Equation 6:[6]

$$\text{ECSA} = \frac{C_{dl}}{C_s} \tag{6}$$

where C_s is the specific capacitance of the sample, or the capacitance of an atomically smooth planar surface of the material per unit area under identical electrolyte conditions. Here, we used for the C_3N_4 containing catalysts and for CDots a general specific capacitance of 0.022 mF cm^{-2} based on typical values reported for carbon electrodes[7] in 5 M KOH solution. For Co_3O_4 and Co_3O_4-CDots we used 0.04 mF cm^{-2} in 1 M KOH solution recommended by reference 7 which is between 0.022 mF cm^{-2} for C and 0.06 mF cm^{-2} used for oxides.

Supplementary Figures

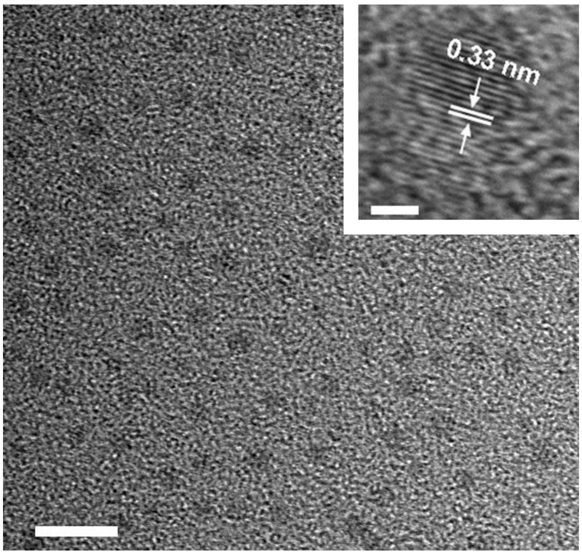

Supplementary Figure 1. The TEM image of CDots and HRTEM image of CDots (inset), scale bar 10 nm and 2 nm (inset).

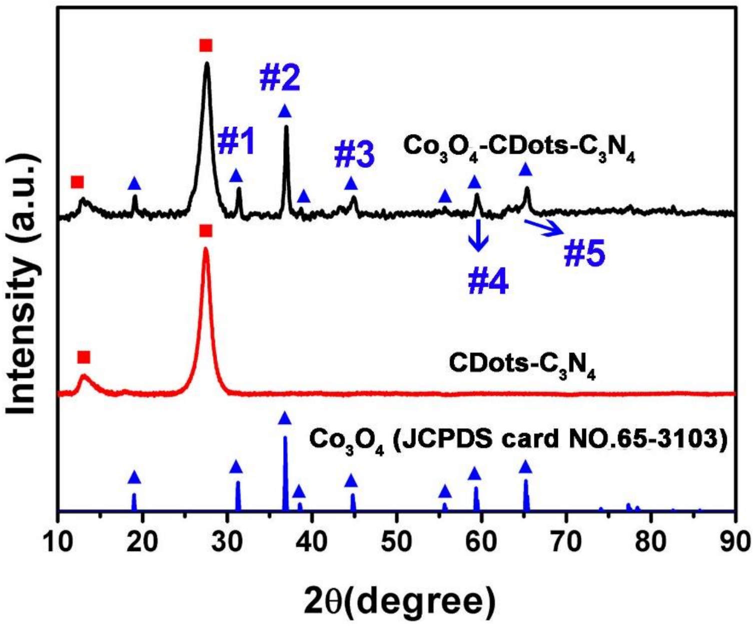

Supplementary Figure 2. Large-angle XRD patterns of CDots-C_3N_4 (the red trace), Co_3O_4-CDots-C_3N_4 (the black trace); The diffraction peaks of Co_3O_4 are indexed to the face-centered cubic $Fd\bar{3}m$ symmetry of spinel-type Co_3O_4 structure with lattice parameter a=0.8056 nm (JCPDS card No.65-3103). The characteristic peak for CDots at around 26° from CDots[9, 10] is not visible, which may due to the low diffraction intensity in composites and/or the diffraction response covered by C_3N_4 diffraction signal.

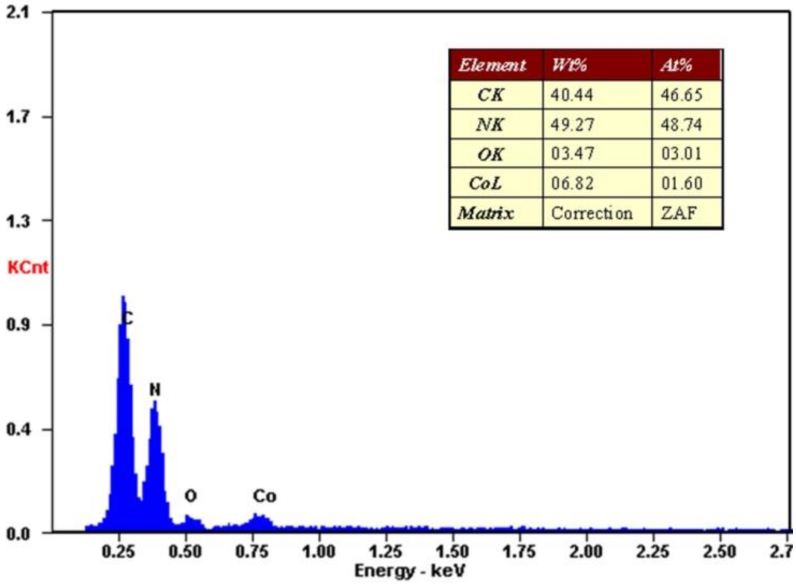

Supplementary Figure 3. The EDX spectrum of Co_3O_4-CDots-C_3N_4.

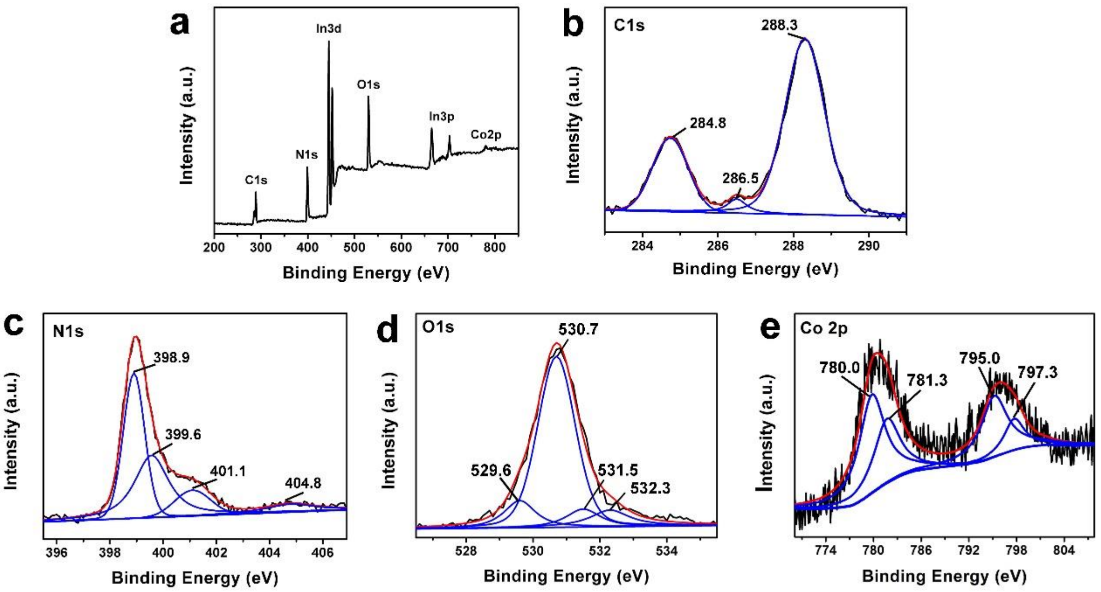

Supplementary Figure 4. XPS spectra of Co_3O_4-CDots-C_3N_4; (a) XPS survey spectrum. The indium signal originates from the substrate on which the sample is put on; The high-resolution XPS spectrum of the (b) C 1s; (c) N 1s; (d) O 1s; (e) Co 2p. Supplementary Fig. 4a shows that Co_3O_4-CDots-C_3N_4 is mainly composed of carbon, nitrogen, oxide and cobalt. As shown in Supplementary Fig. 4b, the C 1s binding energy peaks can be deconvoluted into three different peaks at 284.8, 286.5 and 288.3 eV, corresponding to graphitic carbon, C-O functional groups and N-C=N, respectively.[11] Supplementary Fig. 4c exhibits four peaks at 398.9, 399.6, 401.1 and 404.8 eV that correspond to sp^2 hybridized aromatic N (C=N-C), ertiary N (N-$(C)_3$ or HN-$(C)_2$) and quaternary N (bonding to three carbon atoms in the aromatic cycles), and π excitations.[12] As shown in Fig. 2d, the deconvoluted O 1s spectrum shows four peaks at 529.6, 530.7, 531.5 and 532.3 eV attributed, respectively, to the oxygen species in cobalt oxide, indium oxide, C=O and adsorbed surface water.[13]

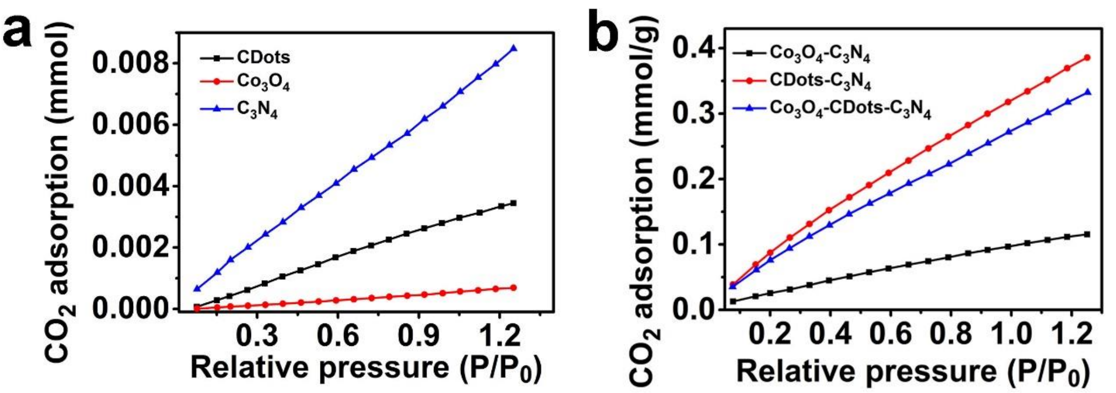

Supplementary Figure 5 CO_2 adsorption on the different components of the catalysts. (a) CO_2 adsorption by 0.001 g CDots, 0.006 g Co_3O_4 and 0.093 g C_3N_4; (b) CO_2 adsorption (mmol $g_{catalyst}^{-1}$) of the composite catalyst. The total CO_2 adsorption of the components in Supplementary Fig. 5a (CO_2(0.001g CDots) + CO_2(0.006 g Co_3O_4) + CO_2(0.093 C_3N_4) = 0.00345 mmol +0.00069 mmol + 0.00848 mmol) is 0.01262 mmol. The adsorption of H^+ by 0.1g Co_3O_4-CDots-C_3N_4 in Supplementary Fig. 5b is 0.0332 mmol. This indicates that the synergistic adsorption of the composite is larger (by a factor of 3) than the individual adsorption of each component.

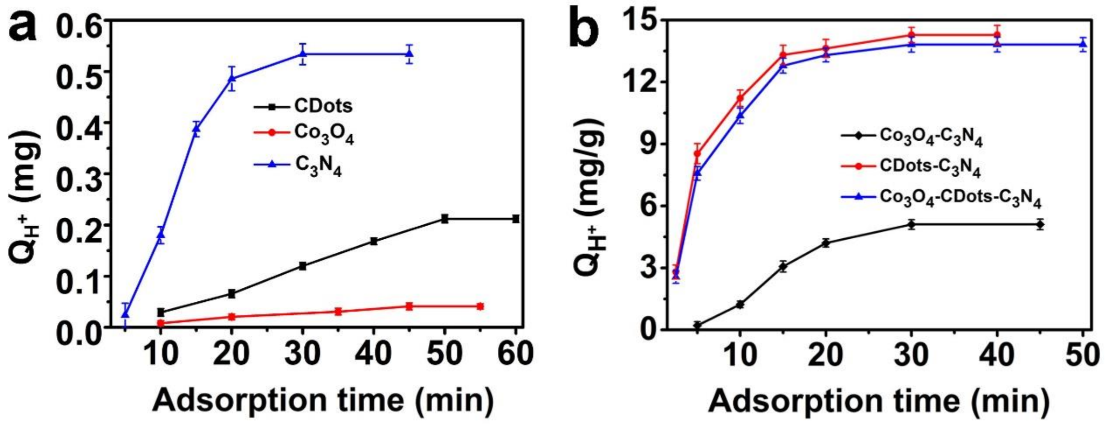

Supplementary Figure 6 H^+ adsorption on the different components of the catalysts. (a) H^+ adsorption by 0.001 g CDots, 0.006 g Co_3O_4 and 0.093 g C_3N_4; (b) H^+ adsorption (mg $g_{catalyst}^{-1}$) of the composite catalyst. The total H^+ adsorption of the components in Supplementary Fig. 6a (H^+(0.001g CDots) + H^+(0.006 g Co_3O_4) + H^+(0.093 C_3N_4) = 0.212 mg + 0.041 mg + 0.534 mg) is 0.787 mg. The adsorption of H^+ by 0.1g Co_3O_4-CDots-C_3N_4 in Supplementary Fig. 6b is 1.38 mg. This indicates that the synergistic adsorption of the composite is larger (by a factor of 2) than the individual

adsorption of each component. Experiments were performed in triplicates and results are shown as mean ± standard deviation.

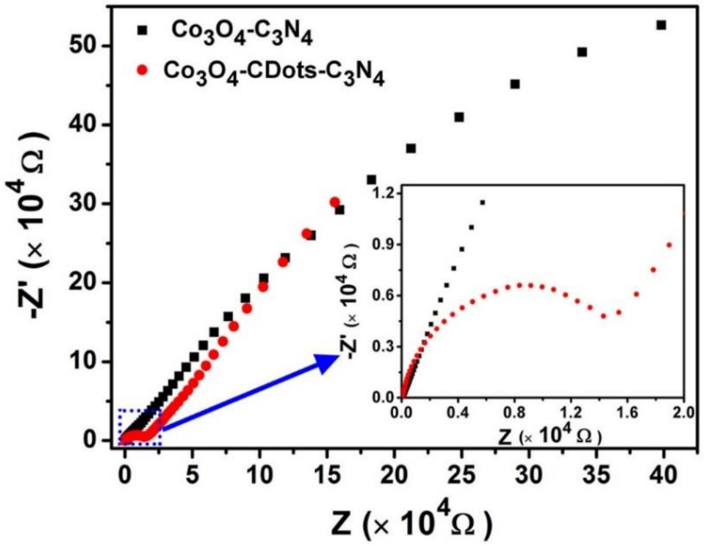

Supplementary Figure 7. Faradaic impedance spectroscopy of Co_3O_4-C_3N_4 and Co_3O_4-CDots-C_3N_4 at overpotential=0.17 V. The Co_3O_4-CDots-C_3N_4 exhibits much smaller Faradaic impedance;

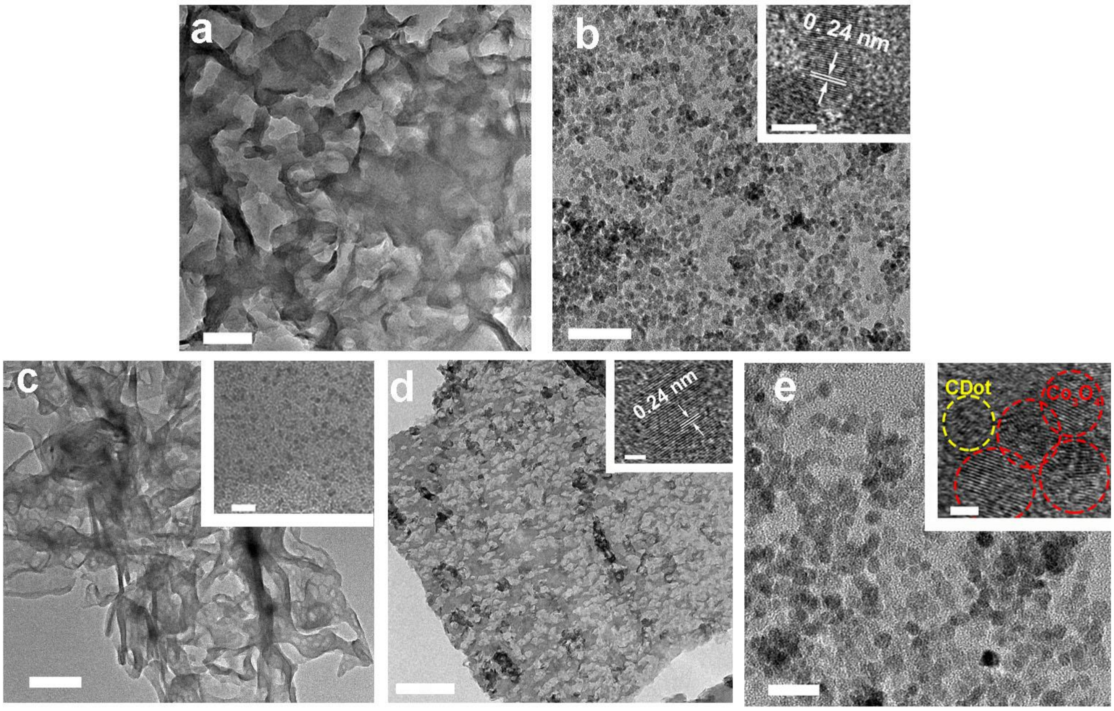

Supplementary Figure 8. (a) The TEM image of a grain of the C_3N_4, scale bar 200 nm; (b)

The TEM image of Co_3O_4 NPs and HRTEM image of Co_3O_4 (inset), scale bar 50 nm and 5 nm (inset); (c) The TEM image of CDots-C_3N_4 and the TEM image of dispersed CDots on C_3N_4 (inset), scale bar 200 nm and 20 nm (inset); (d) The TEM image of a grain of the Co_3O_4-C_3N_4 composite and HRTEM image of Co_3O_4 (inset), scale bar 100 nm and 2 nm (inset); (e) The TEM image of Co_3O_4-CDots and HRTEM image of Co_3O_4-CDots (inset), scale bar 20 nm and 2 nm (inset).

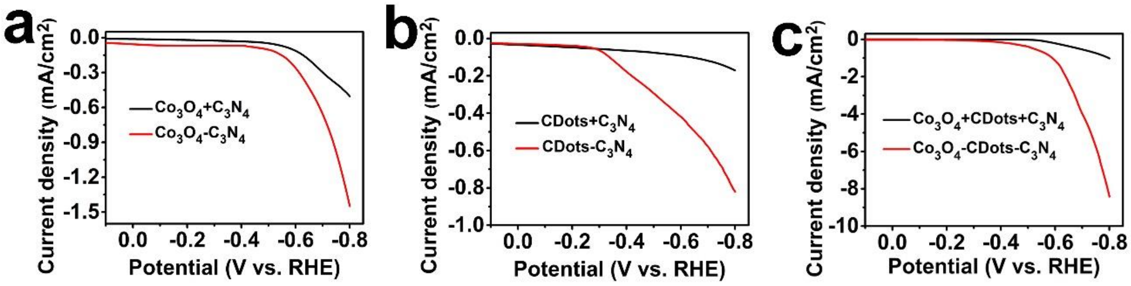

Supplementary Figure 9. (a) LSVs for Co_3O_4+C_3N_4 (black trace) and Co_3O_4-C_3N_4 (red trace) in CO_2-saturated 0.5 M $KHCO_3$ electrolyte, 10 $mV \cdot s^{-1}$; (b) LSVs for CDots+C_3N_4 (black trace) and CDots-C_3N_4 (red trace) in CO_2-saturated 0.5 M $KHCO_3$ electrolyte, 10 $mV \cdot s^{-1}$; (a) LSVs for Co_3O_4+CDots+C_3N_4 (black trace) and Co_3O_4-CDots-C_3N_4 (red trace) in CO_2-saturated 0.5 M $KHCO_3$ electrolyte, 10 $mV \cdot s^{-1}$. Co_3O_4+C_3N_4, CDots+C_3N_4 and Co_3O_4+CDots+C_3N_4 were prepared by simple physical mixing. Co_3O_4-C_3N_4, CDots-C_3N_4, and Co_3O_4-CDots-C_3N_4 denote composites prepared by chemical methods. Note that the catalytic activity of the composites (i.e. current density) is much larger than that of the physical mixtures. We attribute this behavior to the proximity between the active sites (different catalysts) in the (chemically blended) composites. Physical mixing does not provide such proximity between the nanosized mixed catalysts.

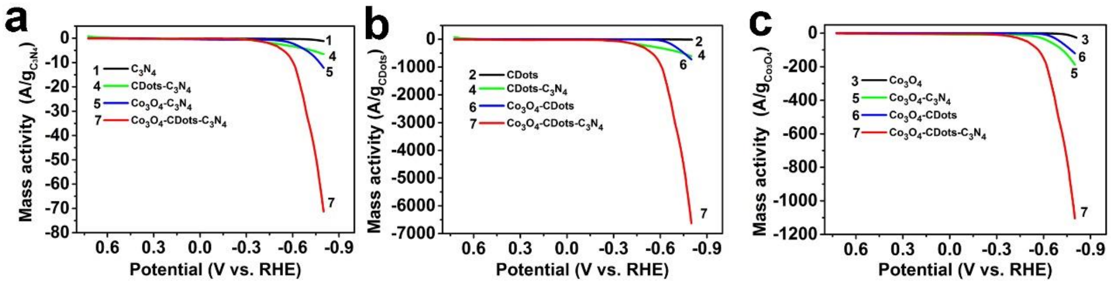

Supplementary Figure 10 The mass activity (current per catalyst mass) of the different

combinations of the catalysts taking into account just one catalyst component denoted in the y axis: (a) Mass activity based on the weight of C_3N_4 in each combination of catalysts; (b) Mass activity based on the weight of CDots in each combination of catalysts; (c) Mass activity based on the weight of Co_3O_4 in each combination of catalysts. Note: The mass activity does not depend on the surface area of the electrode (either real or geometrical). Addition of catalyst components to an initial component increases the mass activity which is optimized in terms of the gas composition (H_2/CO) and current (syngas generation rate) for the three component Co_3O_4-CDots-C_3N_4 catalyst.

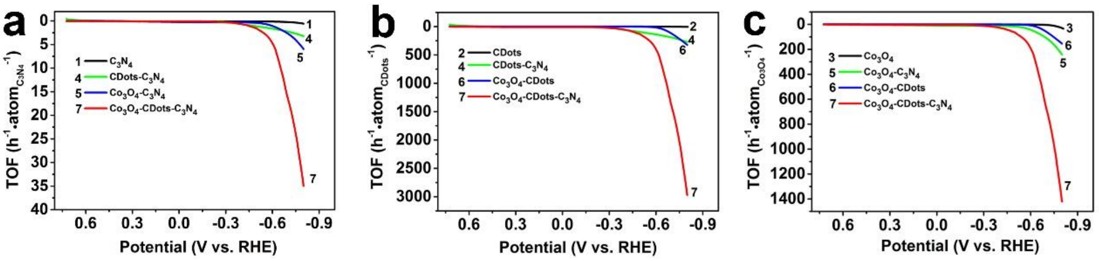

Supplementary Figure 11 The turn over frequency (TOF) of the different combinations of catalysts taking into account the number of atoms in just one component as representing the number of active sites of this component. (a) TOFs based on the number of atoms of C_3N_4 in each combination of catalysts; (b) TOFs based on the number of atoms of CDots in each combination of catalysts; (c) TOFs based on the number of atoms of Co_3O_4 in each combination of catalysts. TOF = (number of reacted electrons per unit time)/(number of catalyst active sites). The number of catalyst active sites was approximated by the number of atoms of a single catalyst component. Note: The TOF does not depend on the surface area of the electrode (either real or geometrical); Addition of catalyst components to an initial component increases the TOF which is optimized in terms of the gas composition (H_2/CO) and current (syngas generation rate) for the three component Co_3O_4-CDots-C_3N_4 catalyst.

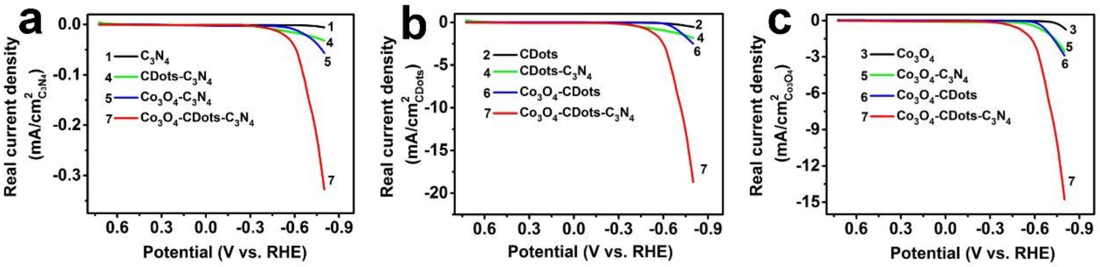

Supplementary Figure 12 The real current density (current per real area of one catalyst component) of the different combinations of the catalysts taking into account the real area of just one catalyst component denoted in the y axis: (a) Real current density based on the ECSA of C_3N_4 in each combination of catalysts; (b) Real current density based on the ECSA of CDots in each combination of catalysts; (c) Real current density based on the ECSA of Co_3O_4 in each combination of catalysts. Note: Addition of catalyst components to an initial component increases the real current density which is optimized in terms of the gas composition (H_2/CO) and current (syngas generation rate) for the three component Co_3O_4-CDots-C_3N_4 catalyst.

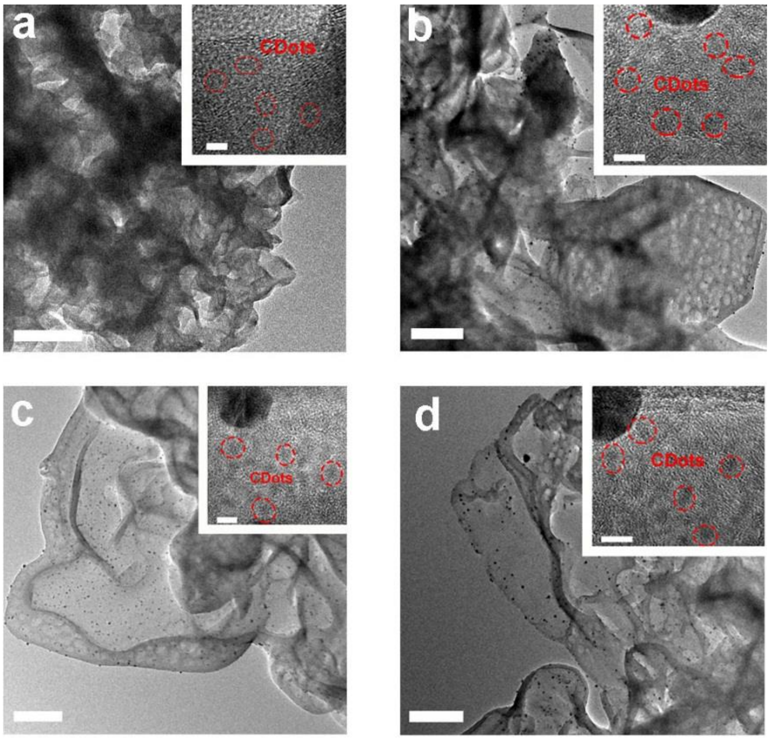

Supplementary Figure 13. The TEM image and HRTEM image (inset) of (a) MoS_2-CDots-C_3N_4, scale bar 100 nm and 5 nm (inset), (b) Pt-CDots-C_3N_4, scale bar 200 nm and 5 nm (inset), (c) Au-CDots-C_3N_4, scale bar 200 nm and 5 nm (inset), (d) h-Au-CDots-C_3N_4, scale bar 200 nm and 5 nm (inset). Typical CDots are circled by red ring.

Supplementary References

[1] Thommes, M., et al. Physisorption of gases, with special reference to the evaluation of surface area and pore size distribution (IUPAC Technical Report). *Pure Appl. Chem.* **87**,

1051-1069 (2015).

[2] Landers, J., Gor, G. Y. & Neimark, A. V. Density functional theory methods for characterization of porous materials. *Colloid. Surfaces A: Physicochem. Eng. Aspects* **437**, 3-32 (2013).

[3] Cychosz, K. A., Nicolas, G. R., Martı´nez, J. G. & Thommes, M. Recent advances in the textural characterization of hierarchically structured nanoporous materials, *Chem. Soc. Rev.* **46**, 389-414 (2017).

[4] ASAP 2020 Accelerated Surface Area and Porosimetry System Operator's Manual, V4.02, 443-451, (2013).

[5] Benck, J. D., Chen, Z., Kuritzky, L. Y., Forman, A. J. & Jaramillo, T. F. Amorphous molybdenum sulfide catalysts for electrochemical hydrogen production: insights into the origin of their catalytic activity, *ACS Catal.* **2**, 1916-1923, (2012).

[6] McCrory, C. C. L., et al. Benchmarking hydrogen evolving reaction and oxygen evolving reaction electrocatalysts for solar water splitting devices, *J. Am. Chem. Soc.* **137**, 4347-4357, (2015).

[7] McCrory, C. C. L., Jung, S., Peters, J. C. & Jaramillo, T. F. Benchmarking heterogeneous electrocatalysts for the oxygen evolution reaction, *J. Am. Chem. Soc.* **135**, 16977-16987 (2013).

[8] Chi B., Lin H., Li J., Wang N. & Yang J., Comparison of three preparation methods of $NiCo_2O_4$ electrodes, *Inter. J. of Hydrogen Energy* **31**, 1210-1214, (2006).

[9] Yan, S. C., Li, Z. S. & Zou, Z. G. Photodegradation performance of g-C_3N_4 fabricated by directly heating melamine. *Langmuir* **25,** 10397-10401 (2009).

[10] Tang, D. et al. Carbon quantum dot/NiFe layered double-hydroxide composite as a highly efficient electrocatalyst for water oxidation. *ACS Appl. Mater. Interfaces,* **6,** 7918-7925 (2014).

[11] Liu, J., et al. Metal-free efficient photocatalyst for stable visible water splitting via a two-electron pathway. *Science* 347, 970-974 (2015).

[12] Khabashesku, V. N., Zimmerman, J. L. & Margrave, J. L. Powder synthesis and characterization of amorphous carbon nitride. *Chem. Mater.* **12,** 3264-3270 (2000).

[13] Guo, Q. et al. Synthesis of carbon nitride nanotubes with the C_3N_4 stoichiometry via a

benzene-thermal process at low temperatures. *Chem. Commun*. **2004**, 26-27 (2004).
